# Medical Treatment of Hyperthyroidism
*A Paper read at the Meeting of the Bristol Medico-Chirurgical Society, on Wednesday, 10th April, 1935.


**Published:** 1935

**Authors:** J. Innes Noble


### MEDICAL TREATMENT OF HYPERTHYROIDISM.*

BY

J. INNES NOBLE, M.B., CH.B., F.R.C.S.

---

MR. PRESIDENT, LADIES AND GENTLEMEN,—It is with some trepidation that I stand here, and I am surprised at my audacity in venturing to speak on the medical treatment of a condition which is so generally dismissed from the physician's field and given over without hesitation to the surgeon. Nevertheless, it is a fact that in the last seven years of my practice I have treated fourteen cases of hyperthyroidism without the aid of the surgeon and with no bad result in these cases.

Let me hastily say that the line of treatment which has been adopted has not been in any way unusual, has not been based on the use of any long-forgotten simple, nor does it introduce the exhibition of any new chemical substance. This short paper will not add anything at all to the bibliography of the disease, not will it stir up any controversy because of its fantastic claims.

The chief reasons I put forward as some plea for

* A Paper read at the Meeting of the Bristol Medico-Chirurgical Society, on Wednesday, 10th April, 1935.

giving a résumé of the treatment of my cases are summarized as follows:—

1. The poor results which I am convinced the present surgical treatment gives.

2. The conviction that there are two main factors in every case of hyperthyroidism which can be dealt with by the physician.

3. The prevailing disinclination of the physician *really* to take charge of a case of hyperthyroidism in a sufficiently thorough way.

It must be the practice of all physicians, whether consultants or humbler lesser fry, to heave a deep sigh when confronted with any case of excess of thyroid secretion. And I believe that here is the first cause of the reputedly unsuccessful treatment medically. It must be admitted that these cases are notoriously irksome. They call for all the patience that the attendant has, and they are exhausting to the doctor and those in contact with the patient.

The essential beginning to medical treatment of the condition is *early diagnosis.* I am sure that the disease is one which is increasing each year in these hectic times, and I have adopted the practice of diagnosing every case in women who have persistent increased frequency of the pulse, loss in weight, and tremor as being hyperthyroidism until I can convince myself that there is some other lesion. I cannot stress too highly the necessity for earliest possible diagnosis.

I have said that there are, in my opinion, two main causes of the condition which have been common to all the cases I have had under observation. These are : (1) Focal sepsis ; (2) mental stress or psychological distress. These will be dealt with fully.

The first essential in the treatment is the segregation of the patient and the choice of a suitable environment.

It is futile, I think, to attempt to treat a case in a household where normal everyday activities go on or in a mixed surgical, medical and obstetrical nursing home. The patient must have a quiet, pleasant, room where she will be looked after by a sensible, restful nurse who will be solely responsible to the physician for all that concerns the patient. It must be a Medic law that no fussy relative or those who wish to cheer the patient from her nervous state will enter the room without the express permission of the physician. As a rule, if the patient is a married woman I exclude all relations except the husband, and he also is warned not to see the patient if it has been ascertained from her that his presence, as it often is, is inimical. The patient is carefully told at the outset that she must be prepared to stay at least three months in bed, that the primary object of her treatment is rest and quiet, and that the disease is due to excess of the secretion of the thyroid gland, which causes her to burn up her body fuel in an intense way. It is explained to her that each movement she makes is burning up tremendous energy. She is allowed to read or be read to, she is permitted to knit or do some not too fine sewing, and to interest herself in the daily happenings which affect her.

In such a way is the patient made to rest in mind and body. She is encouraged to tell any fears she has to the nurse, and every effort is made to give her tangible proof that all is well with anyone she

K

has an interest in. It is found that when the patient is first put to bed in quiet surroundings the pulse-rate falls and she will begin to put on weight.

Attention can now be given to the first of the two main causes which have been common to all my cases, namely the presence of a septic focus.

In twelve of the fourteen cases the focus has been a dental one. The large majority of these cases have shown apical abscesses. Most of the patients have not been guilty of neglect of their teeth. In fact, in two cases a dental overhaul had taken place no later than three weeks prior to my seeing the patient. Their mouths showed as much amalgam as would have accounted for some part of the body weight, and were, no doubt, a great source of pride to the dental surgeon. Skiagraphic examination of the teeth revealed no fewer than nine apical abscesses in one case, and in several cases all remaining teeth were badly infected. The patient's condition usually has warranted immediate progress with the removal of the septic teeth. This was done by stages under gas in most cases in the patient's room, or in some few cases after the administration of paraldehyde per rectum. Where few teeth have been infected the dental surgeon has preferred to make the extraction with local anæsthesia either at interrupted sittings or at a single sitting by regional block anæsthesia.

The immediate progress of the patient after the teeth extractions has been one of some anxiety. Within twelve hours of the extractions the pulse-rate usually is markedly increased in frequency; there is an exacerbation of all symptoms, increase by measurement in the size of the gland, exaggeration of the tremor and extreme perspiration. In no case did the exacerbation last longer than one week after the teeth extraction.

In other two cases under my observation there was frank tonsillar infection. It must not be forgotten that the pharynx is injected in all cases of hyperthyroidism and that the tonsils are swollen. It must be proved that there is genuine infection of the tonsils before recourse is had to tonsillectomy. Tonsillectomy was actually performed in the two cases, and was followed by the exacerbation described after the dental treatment.

The stage has now been reached where the first of the two factors common to all cases has been dealt with, namely focal sepsis. We are now able to deal with the second common factor, which I wish to emphasize to the full.

In every case I have found some striking history of mental strain or psychological disturbance. By far the commonest was that of difficulty in marital relationships, and was described as sexual excitation without orgasm. This repeated nervous strain without adequate satisfaction to the impulses produced was found to be disastrous to the patient's nervous system as a whole, and more particularly to the mechanism of the thyroid gland. The placing of the patient beyond the possibility of such sexual excitement was found to have a rapidly beneficial effect, and later, on her return to family life, careful discussion of the subject with both the woman and her husband was entered into with happy results in these cases.

I am well aware that the state of affairs I have mentioned is extremely common in the married state, and I am not contending that every married woman who suffers from lack of sex knowledge will develop excess of thyroid secretion, but I do wish to stress the presence of this unhappy state in several of my cases. The ignorance in this country even in these more sensibly enlightened days on the requirements of the sexual life of the female is amazing. We find more enlightenment in the European and Latin countries, but we are apt to put this knowledge down to not-nice-mindedness and lack of due restraint. It is usually a simple matter to correct the ignorance of the husband of a patient with extremely happy results.

Other psychological factors were found in the series of cases as follows:—

Two cases were in young married women who began their married lives in the parental home. One of these said that she had no privacy, and that she felt she was being spied upon.

Two cases were in unmarried women. In one case the girl had been engaged to be married for a long time and feared pregnancy. In one other there was a history of perversion of sex with another female.

Two cases were married women in the pre-climacteric state where unfortunately aloofness had developed. Explanation of the phenomenon to the husband was necessary.

In other cases one member of a family or one relative, usually a mother-in-law or aunt, was found to be a source of continual irritation and even intense antipathy to the patient.

Extreme tact is necessary in dealing with the situation, but firmness must be exercised in excluding the inimical person from proximity of the patient. This part of the treatment will not be easy to most physicians, for the chief reason that he is himself ignorant on the sexual question it raises, and also because he may allow himself to be brow-beaten by some domineering relative who is convinced that the patient is suffering from "nerves"; but if any success is to be gained from medical treatment the question of the psychological aspect must be very thoroughly gone into. In some cases the patient's husband will resent discussion of his marital relations, and the physician will be accused of prying into precincts which are none of his business.

The fourth essential is the patient's intake of food. It is explained at the outset, as has been said, that the patient burns up her body fuel. It is further explained to the patient that the body is like a furnace in the disease and requires stoking frequently and plentifully. There is no ideal dietary. The patient must be given three good balanced meals a day, and care should be taken to quash the belief that when she is in bed she does not need as much food as if she were getting about. Special care is taken to give abundance of sugar and carbohydrate generally in any form the patient likes, and large quantities of jam, honey and glucose are encouraged. This essential in the conduct of the case requires a good nurse, and she must encourage the patient to eat when the appetite is flagging, or when, strangely enough, as I have found in some of my cases, the modern patient begins to be convinced she is getting too fat.

The fifth principle in the treatment is the exhibition of drugs. I have purposely rated this down in the list of essential headings.

It will be found that the early excitability of the patient calls for some sedative. Most of the cases were given luminal gr. 1 t.i.d., but lately I have exhibited prominal, one tablet twice daily, and this has not been characterized by the depression which luminal gives. The prominal was given after breakfast and after tea. Hebaral sodium, gr. 3, in capsule form was found to be very suitable in one case. In the very early stages sleep is usually disturbed or difficult. The best hypnotic was found to be paraldehyde in a nightly dose of 2 drms.

The last essential of the treatment which I have postulated is iodine therapy.

I do not begin this until the patient has been in bed for two weeks. Iodine is then given for ten days in the form of Lugol's solution m. 10 t.i.d. It is observed that about the tenth day the gland becomes harder and more vascular, and the dose of the iodine solution is diminished to m. 5 t.i.d., and if weight is gained at a satisfactory rate again reduced after a month to m. 5 b.i.d. I have found that it is wise to increase the dose to the original m. 10 t.i.d. on the two days preceding and during the menstrual period.

When the weight has increased to considerably more than the highest weight ever previously reached by the patient, and the pulse-rate has fallen to eighty or below, I allow her to sit up in the room for one hour daily, and at the same time gentle massage is given to the limbs for ten minutes only at a time. Careful record of the pulse-rate is made before and after the movements. If weight has been maintained and the pulse-rate has not become increased one more hour is allowed per day in each successive week. The patient is also encouraged to walk about the room.

This very critical period may occupy as long as two months, until the patient takes short drives and then walks out of doors. Special care is taken to watch the progress of the patient during this stage at the menstrual epochs. It may be necessary to curtail the patient's activities at these times. It will be noted by the physician that there is always an exacerbation of symptoms at these epochs.

Other points which are worthy of special note during the treatment are as follows:—

1. Careful weekly weighing is essential, but daily weighing is too worrying to the patient, who begins to be too interested in this procedure.

2. Careful records of the pulse must be made at 10 a.m. and 8 p.m.

3. Measurement of the neck is made by the physician weekly.

4. Visits by the physician should be made preferably in the evening, when he sees the patient at her worst after the ennui of the day. These visits should be short, not by appointment, and should be paid only weekly unless special summons is made by the nurse.

5. If the patient rebels at the amount of food she is asked to take it is better to leave out two meals in one day, substituting glucose and barley-water than to persist with the food or to lessen the amount of the daily meals.

6. Very careful care of the bowel and skin is necessary. Perspiration in these cases is most profuse, and powdering of the skin generally is frequently necessary.

7. Insist on censoring the patient's incoming correspondence, and warn all relatives that she must not be disturbed by untoward happenings in the family acquaintance.

8. Always be encouraging in look and speech to the patient.

It will be seen that there is nothing new in the description of the medical treatment I have given except perhaps the stress I have laid on the psychological aspect of the disease.

It will be argued by the surgeon that after the treatment given the patient will now be adequately prepared for his attention. The cases I have had under observation, however, have not shown any backward tendency, and I have made excerpts from the case histories of five of these who have been observed for five years.

**Case 1.**—Miss H. C. G., aged 40 when first seen in 1930. Intense dental infection. Brother subject to fits of mental excitement and patient feared violence. Abnormal sex relations with other woman. Weighed 6 st. 7 lb. Seen 28th March, 1935. Weight 7 st. 9 lb. After running down stairs pulse 80. Does art work for long hours and guide work.

**Case 2.**—Mrs. W. S., aged 43 when seen in 1929. Dental infection, apical abscesses. Psychological factor. Early menopause, aloofness. Weight 7 st. Seen 27th March, 1935. Weight 8 st. 2 lb. Pulse 88. Walks and does own household work.

**Case 3.**—Mrs. B. L., aged 40 when seen in 1930. Weight 10 st. 1 lb. Intense dental infection. All teeth removed. Husband's death. Early menopause. Pulse 140. Seen 28th March, 1935. Weight 11 st. 10 lb. Pulse 78. Does own house work, etc.

**Case 4.**—Mrs. P. F., aged 34 when seen in 1930. Pulse 130. Weight 6 st. 10 lb. Tonsillar infection removed. Psychological factor. Marital incompatibility due to male ignorance. Seen 2nd April, 1935. Pulse 80. Weight 8 st. 4 lb. Withstood operation for adherent omentum, falling through greenhouse before that. Does own housework with aid of daily woman.

**Case 5.**—Miss V. V., aged 25. Seen 1929. Weight 7 st. 8 lb. Dental infection. Engaged to be married. Feared pregnancy. Seen 20th March, 1935. Weight 8 st. 7 lb. One child. Pulse 80.

The remaining cases were first seen less than five years ago, but none is unsatisfactory.

In conclusion, I am fully aware that all I have said about this disease, with the possible exception of the psychological factor, is admitted by physicians and surgeons alike as being a prelude to the operative treatment. It may be that I have been lucky in the choice of my cases, and it may be that in time manifestations of acute exacerbation may occur in these cases. It may be that the success in the treatment of the cases has been an act of God, as was suggested to me the other day by a distinguished physician now present. These are the facts of the cases nevertheless, and I am persuaded by these results that hyperthyroidism falls within the scope of the physician alone.

In those cases where by failure of early diagnosis or by inadequacy of medical treatment the patient's condition is found to be extremely grave I admit that surgery must be resorted to as a means of urgency to save the patient's life ; but I am equally convinced that in time the condition will again be left to the physician, possibly because of some hitherto unrecognized factor in the pathology of the disease or by some fresh line of treatment.

